# Insight into the Potential of Somatostatin Vaccination with Goats as a Model: From a Perspective of the Gastrointestinal Microbiota

**DOI:** 10.3390/ani15050728

**Published:** 2025-03-04

**Authors:** Xiaoli Zhang, Juncai Chen, Siqi Zhang, Bingni Wei, Yanguo Han, Zhongquan Zhao

**Affiliations:** Chongqing Key Laboratory of Herbivore Science, College of Animal Science and Technology, Southwest University, Chongqing 400715, China; zhangxiaoli826@swu.edu.cn (X.Z.);

**Keywords:** fermentation, gastrointestinal microbiota, goat, somatostatin vaccination

## Abstract

Deciphering the gastrointestinal microbial response to oral SS DNA vaccines with different doses is helpful for identifying the mechanism for effective utilization of the vaccine for improving animal production. Here, we conduct a comparative study with different doses of vaccine (control: empty plasmid; low dose: 1 × 10^7^ CFU vaccine; high dose: 1 × 10^12^ CFU vaccine) using goat as a case to investigate the potential of somatostatin vaccination from the entire gastrointestinal microbiota perspective. Our results show that body weight gain and slaughter rate are greater in the L_SS group than in the C_SS group. Compared with the C_SS group, the GH concentration is reduced, while the SS concentration is elevated in the cecum of L_SS goats. Moreover, the SCFAs concentration is elevated in the L_SS goats, the acetate molar proportion is lower in the rumen, the proportion of the acetate is decreased, and propionate is increased in the cecum of L_SS goats. Our data indicate that the low-dose somatostatin vaccine possesses a more efficient improvement in the productivity of goats, emphasizing that the dosage should be considered to reach its optimal effect on the host. Moreover, we find that different doses of the SS vaccination select distinct microbial communities in the gastrointestinal tract. Beta diversity analysis shows a significant interaction. Microorganisms capable of converting nutrients, including *Ruminococcacease*, *Butyrivibrio*, *Akkermansia*, and *Lachnospiraceae* are enriched, altering the gastrointestinal fermentation response to SS DNA vaccination of ruminants. Moreover, the correlation analysis results revealing these biomarkers have a close association with the phenotypes of productivity. These results imply that somatostatin immunoneutralization might directly alter the gastrointestinal tract commensal bacterial structure, improving gastrointestinal homeostasis, and, thus, modifying the fermentability and effected hormone level to improve the productivity of goats. Our study extends the understanding of the somatostatin vaccine regulation of ruminants’ growth through the entire gastrointestinal microbial perspective.

## Simple Summary

Active immunization of exogenous somatostatin can effectively neutralize endogenous somatostatin and promote animal production. Somatostatin is also well known as a peptide distributed widely throughout the gut, as one of the hormones of the gastrointestinal tract. This research evaluates the efficacy of the somatostatin immunoneutralization on growth, hormone level, and gastrointestinal tract microbiota of goat, as well as the relationship between those phenotypes and the functional microorganisms with the use of vaccines. The data indicate that the low-dose somatostatin vaccine possesses a more efficient route for improving the productivity of goats, emphasizing that the dosage should be considered in order to reach its optimal effect on the host. Of the utmost importance, microorganisms capable of converting nutrients were enriched, altering the gastrointestinal fermentation response to low-dose somatostatin vaccination of ruminants. Overall, low-dose somatostatin vaccination altered the composition of gastrointestinal microbiota and promoted animal production.

## 1. Introduction

Goats, as a typical and common ruminant, are distinguishable from monogastric animals due to their capacity to convert plant cell fibers into high-quality animal products [1], such as healthy unsaturated fatty acids and bioactive phospholipids [2,3]. Consequently, the production of goat products consumes fewer grain resources [4] in a world of finite biological resources [5]. Of particular interest, goats possess the advantages of roughage resistance and suitability for small-scale agricultural operations as well as enhanced tolerance to poor environmental conditions [6]. Since the late twentieth century, experts and scholars have made a lot of efforts, including extensive breeding and management manipulation, to enhance production performance and increase their economic and social value [7]. Hormones derived from the hypothalamus, especially the GH-IGF-1 (insulin-like growth factor 1) axis in the anterior pituitary, play a core role in animal productivity [8]. Therefore, research into hormonal regulators that improve the productive performance of animals has been regarded as one of the prominent fields worldwide, particularly regarding substances that disinhibit and neutralize statins.

As its name suggests, somatostatin (SS) is well known for its regulatory effects on growth hormones [9], and has recently received attention for its role in the regulation of GH-IGF-1 axis secretion in altering animal production [10]. Previous studies suggested that active immunization of exogenous SS can effectively neutralize endogenous SS and promote animal production [11]. Concretely, the oral SS DNA vaccine is attributable to improving the growth performance of animals through an influence on GH and SS secretion [10]. These studies indicate the feasibility and effectiveness of enhancing animal productivity by oral SS DNA vaccine. Of note, somatostatin is also well known as a peptide distributed widely throughout the gut, one of the hormones of the GIT [12], inhibiting a variety of gastrointestinal functions that may influence the activity of the gastrointestinal microbiota [13]. It is hypothesized that the SS DNA vaccine could alter hormones and select distinct functional microbiomes, which might modulate the performance of the host. Previous studies have demonstrated that the capacity of somatostatin is dependent on biologically active forms, doses, and vaccination intervals [14,15,16,17]. Undoubtedly, a comprehensive understanding of the gastrointestinal microbial response to oral SS DNA vaccine is beneficial for identifying the mechanism for effective utilization of the vaccine to improve animal production. However, the gastrointestinal microbial response to oral SS DNA vaccines at different doses is still an unsolved mystery. To address this knowledge gap, we conduct a comparative study with different doses of vaccine, using goat as a case, to investigate the potential of somatostatin vaccination from a gastrointestinal microbiota perspective.

## 2. Materials and Methods

### 2.1. Animals and Experimental Design

**Figure 1:**
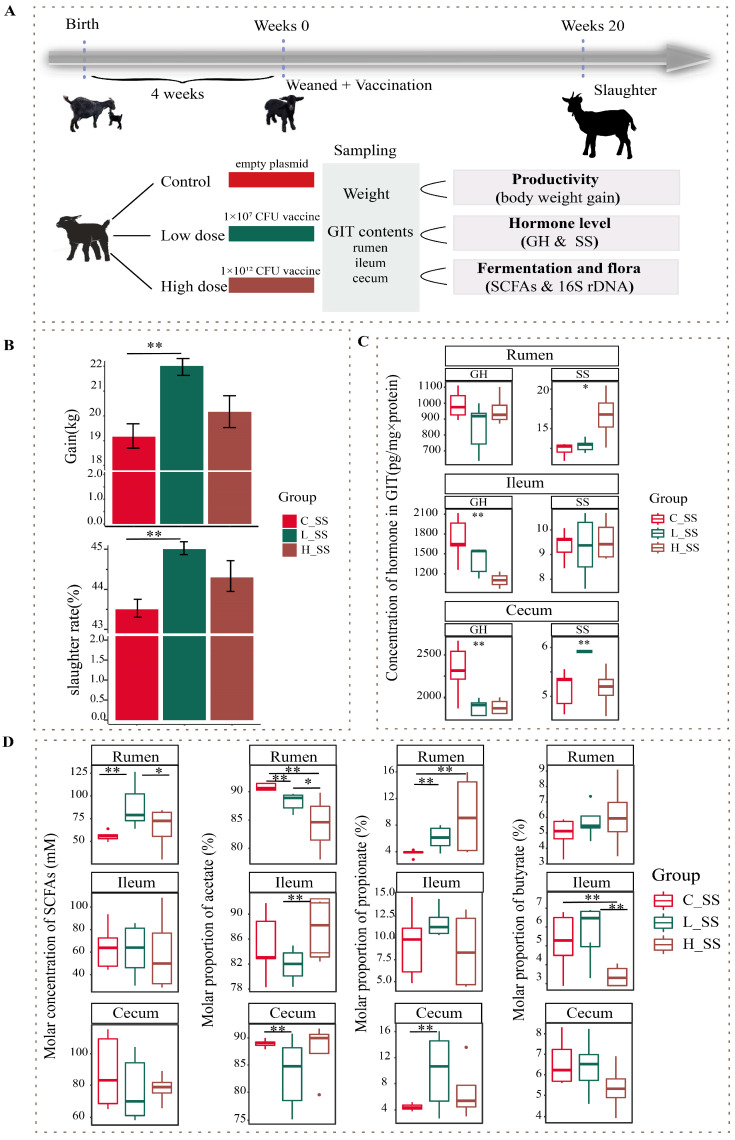
The somatostatin vaccination altering the phenotype of productivity in goats. (**A**) Overview of the design of the experiment and sample collection. (**B**) The profile of body weight gain in goat vaccinated with different doses of somatostatin vaccine. (**C**) The GH and SS concentration of gastrointestinal contents among three groups. (**D**) The somatostatin vaccination altering the fermentation types of gastrointestinal tract in goats. C_SS: control group; L_SS: low-dose group; H_SS: high-dose group. Asterisks denote significant *p* values: NS. *p* > 0.05, * *p* < 0.05, ** *p* < 0.01.

Fifteen healthy weaned DaZhu black goats (a native breed in Chongqing Province, China) were used in this experiment. Weaned goats were randomly allocated to the negative control group (C_SS), the low-dose SS DNA vaccine group (L_SS), and the high-dose SS DNA vaccine group (H_SS). All goats were initially administered 10 mL of sodium bicarbonate solution (7.5%) orally 30 min prior to immunization, followed by oral administration of empty plasmid (pVAX-asd, C_SS), low-dose SS DNA vaccine (ptCS/2SS-asd, 1 × 10^7^ CFU, L_SS), and high-dose SS DNA vaccine (ptCS/2SS-asd, 1 × 10^12^ CFU, H_SS) dissolved in 10 mL sterile saline solution at weeks 0, 4, 8, and 16 of the study period, respectively. The orally administered SS DNA vaccine was constructed by Chongqing Engineering Research Centre for Herbivores Resource Protection and Utilization, and its safety and application were kindly provided by Han Yanguo [17]. Specifically, the HBsAg-S-2SS fusion gene (two copies of the somatostatin gene were inserted into the hepatitis B surface antigen S gene), the tPA signal peptide, and CpG adjuvant were inserted into the pVAX1-asd vector and subsequently electroporated into the mutant attenuated *Salmonella typhimurium X9241* with deletion of the asd and crp genes (CFU is designated as a unit of dose). The *Salmonella typhimurium X9241* strain was generously provided by Dr. Qingke Kong from Southwest University. The animals were housed in individual pens with ad libitum access to water and fed a total mixed ration diet, composing 40% fresh grass and 60% concentrate, twice per day at 8:00 and 17:00. The concentrates ingredients included 56.7% maize flour, 13.3% soybean meal, 14.2% wheat bran, 6.2% fat powder, 1.5% calcium carbonate, 2.0% calcium hydro-phosphate, 1.5% carbamide, 1.0% salt, and a 3.6% mineral and vitamin premix. The experimental period lasted for 20 weeks. A schematic of animal experiment protocols is depicted in Figure 1A.

### 2.2. Sample Collection

Goats were euthanized via exsanguination from the jugular vein following anesthesia induced by intravenous administration of sodium pentobarbital solution (30 mg/kg body weight) at the end of the experimental period. The rumen samples were collected from five locations in the rumen (the anterior dorsal, anterior ventral, medium ventral, posterior dorsal, and posterior ventral locations), and combined to represent a homogenous sample [18]. Approximately 1.5 mL of rumen content was placed in a 2 mL sterilized centrifuge tube and stored at −80 °C after freezing in liquid nitrogen. Another sample (about 2 mL rumen fluid), filtered through four layers of polyester monofilament fabric, was acidified with 200 μL 25 g/100 mL metaphosphoric acid and stored at −20 °C until analysis.

The ileal and cecal content samples were collected from the middle region of the respective intestine [19] and separated into two parts, respectively. A subsample (approximately 2 g) of content was placed into a 2 mL sterilized centrifuge tube, frozen in liquid nitrogen immediately, and then subsequently at −80 °C for microbial quantification. Another subsample (1.5 g) was homogenized with an equal volume saline solution by continuous vertexing overnight. Subsequently, the homogenate was centrifuged at 12,000 r/min at 4 °C for 10 min. The supernatants were mixed with a one-tenth volume of 25 g/100 mL metaphosphoric acid, transferred into a new 2.0 mL microcentrifuge tube, and stored at −20 °C for short-chain fatty acids analysis.

### 2.3. Determination of SS Antibody and Hormone Assays by Radioimmunoassay

An indirect enzyme-linked immunosorbent assay was utilized to detect specific SS antibody titers in the goat serum. For detailed steps, refer to Han et al. [17]. Briefly, 96-well plates were coated with 100 ng/100 µL SS in each well. Serum samples (100 µL) were diluted with PBS with tween (1:25, 1:50, 1:100, 1:200, 1:400, 1:800, 1:1600, 1:3200). Endpoint titers were determined as the reciprocal of the highest serum dilution, where the absorbance was greater than the mean plus two standard deviations of the negative control sample (serum samples from all pre-immunized individuals) at the same dilution and the absorbance was greater than or equal to 0.2.

The profiles of somatostatin (SS) and growth hormone (GH) in the GIT were detected by radioimmunoassay (Beijing Sino-UK Institute of Biological Technology, Beijing, China), which have been described in detail previously [10]. The intra-assay and inter-assay coefficients of variation were less than 15%.

### 2.4. Short-Chain Fatty Acids Detected by Liquid Chromatography

To analyze the concentration of short-chain fatty acids (SCFAs, including acetate, propionate, and butyrate) in the rumen, ileum, and cecum, frozen samples (2 mL) were thawed and then centrifuged at 15,000× *g*, at 4 °C, for 10 min in a temperature-controlled centrifuge. Approximately 1 mL supernatant was filtered using a 0.22 μm syringe filter and transferred into a 1.5 mL glass chromatograph vial, and analyzed for SCFAs with a gas chromatograph (GC7890A, Agilent, Santa Clara, CA, USA) equipped with an R flame ionization detector and a DB-FFAP column according to the method described by a previous study [20].

### 2.5. DNA Extraction

Total DNA was extracted from the ruminal, ileal, and cecal digesta samples using the QIAamp^®^ Fast DNA Stool Mini Kit (Qiagen, Dusseldorf, Germany) according to the manufacturer’s instructions with a slight modification. The fluid was incubated at 95 °C instead of the original 70 °C for 10 min after the addition of ASL buffer to lyse both Gram-positive and Gram-negative microbial cells [21]. The resultant quantity of DNA was measured on the Qubit fluorometer using the Qubit TM dsDNA BR Assay/RNA HS Assay (Thermo Fisher Scientific, Waltham, MA, USA). The quality and quantity of DNA were measured based on absorbance at 260 and 280 nm using a NanoDrop ND1000 (NanoDrop Technologies, Inc., Wilmington, DE, USA). The extracted DNA was stored at −20 °C until analyzed.

### 2.6. Amplicon Sequencing and Bioinformatics Analysis

For each extracted genomic DNA sample, the full-length 16S rRNA gene was amplified in triplicate using the universal primers F27 (5′-AGAGTTTGATCMTGGCTCAG-3′) and 1492R (5′-ACCTTGTTACGACTT-3′) [22]. The amplicon products were purified using a QIAquick Gel extraction Kit (Qiagen, Hilden, Germany) [19]. The amplicon library was constructed using the SMRT Bell TM template prep kit 3.0 (Pacific Biosciences, Menlo Park, California, USA) following the manufacturer’s guidelines. Sequencing was performed according to JGI’s standard procedures using the PacBio sequel II platform [23].

Amplicon sequences for bacterial diversity and composition were analyzed according to the method described by previous study [1]. Briefly, the ASVs was clustered by USEARCH (v. 11.0.667) [24] with the method of unoise3 based on the reference database of SILVA (v. 132). The taxonomy was obtained using the RDP classifier (v. 16) assignment with a 0.80 confidence threshold. Alpha and beta diversities were carried out on R (v. 4.1.3) using the package vegan (v. 2.6.2) [25]. To disclose the metergasis of microbiota in GIT by somatostatin vaccination, the sequences were predicted using the PICRUSt2 [26].

### 2.7. Statistical Analysis and Data Visualization

All statistical analysis and image visualization were carried out in R software (v. 4.0.4). The data of phenotype traits, including the growth performance, serum hormone, and SCFAs profiles, among the three groups were checked for normality and analyzed using one-way ANOVA, allowing for equal or unequal variance. These data are presented as medians with interquartile range and visualization using the ggplot2 package [27] in R.

Alpha diversities including Shannon index, richness index, and Simpson index among groups that did not conform to the normal distribution were analyzed using the Wilcox test. Ordination analyses of Bray–Curtis distance were visualized using principal coordinate analysis and assessed using the PERMANONA with 999 permutations. Taxonomic profiles of GIT microbiome were compared among groups using the limma package in R (v. 4.1.3). Significant difference was defined as *p*  <  0.05.

The Spearman correlation coefficients were calculated to evaluate correlations between the dominate different bacteria genera and functional profile. We considered relationships with the criteria of absolute correlation coefficients greater than 0.5 and *p* values less than 0.05 as significant. These data are presented as heatmaps and visualized using the LinkET package [28] and ComplexHeatmap package [29] in R.

## 3. Results

### 3.1. Anti-Somatostatin Antibody Response and Hormone Level as Well as Body Weight Gain Altered by Immunoneutralization SS

The profile of productivity of goats vaccinated with different doses of the SS DNA vaccine were revealed by weight gain and slaughter rate in this study (Figure 1B). Concretely, there were no significant differences in the body weight gain and slaughter rate between the H_SS group and C_SS group, but they were greater in the L_SS group than those of the C_SS group (*p* < 0.05). The anti-SS antibody titers in the low-dose treatment group were greatest at week 8 of the study, which was significantly greater than that at other weeks of the study. These antibody titers in the low-dose treatment group at week 8 of the study were significantly greater than in the other doses of the treatment groups and control groups (Figure S1, *p* < 0.05). Moreover, there was a significant interaction between SS vaccine treatment and measurement time on serum hormone level (Figure S2, *p* < 0.05).

As illustrated in Figure 1C, the concentration of SS in the rumen was significantly affected by different doses of the SS vaccine, and greater in the H_SS group than in the C_SS group (*p* < 0.05). In the ileum, the concentration of GH was highest in the C_SS group followed by the L_SS group (*p* < 0.05). Intriguingly, as contrasted to the C_SS group, the GH concentration was reduced, while the SS concentration was elevated, in the cecum of L_SS goats (*p* < 0.05).

### 3.2. Fermentation Pattern of GIT Was Altered by SS Vaccine with a Dose- and Region-Dependent Manner

To evaluate the fermentation of the GIT among three treatments, we measured the concentrations of acetate, propionate, and butyrate acid in the rumen, the ileum (fore intestine), and the cecum (hind intestine). In contrast to the C_SS group, SCFAs concentration was elevated in the L_SS goats, and acetate molar proportion was lower in the rumen (Figure 1D, *p* < 0.05), while, for the ileum, the fermentation profiles had no difference between the C_SS and L_SS goats (*p* > 0.05), but the acetate molar proportion was lower and the butyrate molar proportion was greater in the H_SS than that in the L_SS (*p* < 0.05). Similarly, the proportion of the acetate was decreased and the propionate was increased in the cecum of L_SS goats when compared with C_SS goats (*p* < 0.05).

### 3.3. The Different Dose SS Vaccination Selected Distinct Microbial Communities in GIT

**Figure 2:**
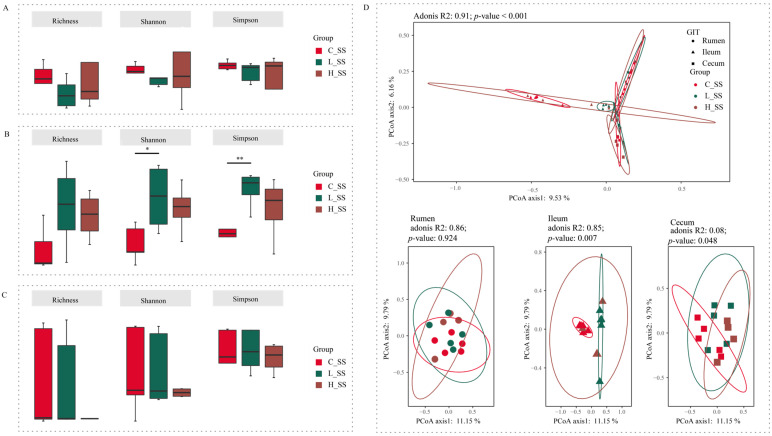
The diversity profiles in GIT of goats for different doses of the somatostatin vaccine. (**A**) Alpha indexes of ASV profiles in the rumen. (**B**) Alpha diversity indexes of ASV profiles in the ileum. (**C**) Alpha diversity indexes of ASV profiles in the cecum. (**D**) Principal coordinate analysis profile of ASV profiles among three groups in all GIT regions (**top**) and individual GIT region (**bottom**). C_SS: control group; L_SS: low-dose group; H_SS: high-dose group. Asterisks denote significant *p* values: NS (non-statistical significance) *p* > 0.05, * *p* < 0.05, ** *p* < 0.01.

Forty-five content samples covering three gastrointestinal regions (rumen, ileum, and cecum) from 15 goats among three groups were analyzed to explore the microbial mechanisms of different doses of the SS vaccine through the GIT. Initial analyses of species richness (richness index) and sample diversity (Shannon and Simpson indexes) for ruminal and cecal microbiota showed that no significant effects were observed for these three alpha indices (Figure 2A,C, *p* > 0.05). Meanwhile, there were no differences between the H_SS group and the L_SS group in ileal alpha indices (Figure 2B, *p* > 0.05). Of note, in the ileum, the L_SS group had greater species diversity when compared with the C_SS group (Figure 2B, *p* < 0.05).

The beta diversity analysis results showed a significant interaction (Figure 2D, *p* < 0.001) between region and treatments on the structure of gastrointestinal microbiota based on the adonis test. In the rumen, the SS vaccination did not alter the microbiome configurations extensively (*p* = 0.924). Intriguingly, each group harbored its unique bacteria structure in the ileum (*p* = 0.007). Synchronously, the cecal microbiota in the L_SS group was remarkably distinct from those in the C_SS and H_SS groups (*p* = 0.048).

### 3.4. The Discriminative Microbiota Among Different-Dose SS Vaccination Groups in GIT

**Figure 3:**
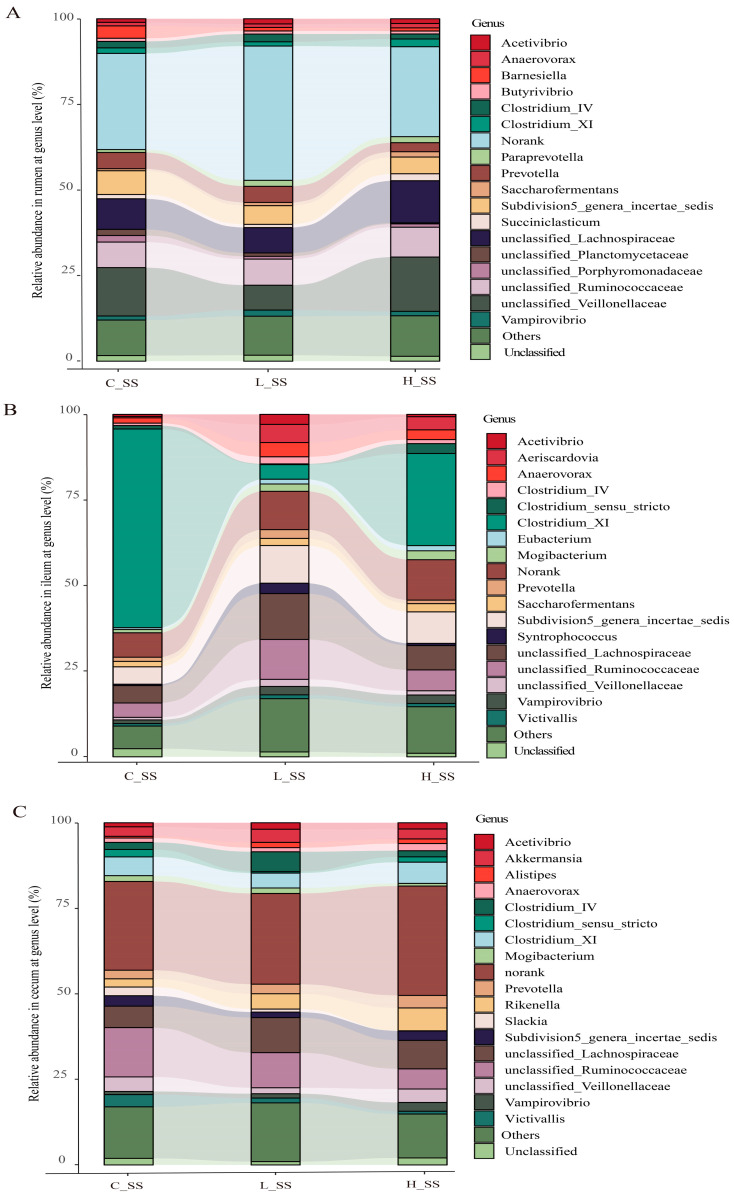
The profile of dominant genus and the microbial biomarkers in GIT of goats vaccinated with different doses of somatostatin. (**A**) Relative abundance in the rumen at genus level. (**B**) Relative abundance in the ileum at genus level. (**C**) Relative abundance in the cecum at genus level. C_SS: control group; L_SS: low-dose group; H_SS: high-dose group.

Correlation analysis revealed that Shannon and Simpson diversity indices were positively correlated with specific bacterial taxa (Figure S3). The composition of gastrointestinal microorganisms varied among the groups at the family level (Figure S4). Microbial taxon classification and Wilcoxon rank-sum test were used to determine the relative proportions of the genus level of dominant taxa and the distinctive microbiota in the GIT of goat vaccinated with different doses of the SS vaccine. Considerable variation was noticed in the composition of flora among three groups in each region (Figure 3). Specifically, the top five dominant genera were *unclassified_Lachnospiraceae* (8.3%~17.2%), *unclassified_Veillonellaceae* (3.9%~12.9%), *unclassified_Ruminococcaceae* (7.8%~10.8%), *Subdivision5_genera_incertae_sedis* (5.7%~7.8%), and *Prevotella* (2.4%~3.1%) in the rumen, in spite of a large proportion of sequences (31.3%~41.1%) remaining unclassified or not ranked (Figure 3A). Abundances of *unclassified_Veillonellaceae* were decreased (*p* < 0.05), while those of *unclassified_Ruminococcaceae* were increased (*p* < 0.05) in the L_SS group when compared with the C_SS group. Intriguingly, the abundance of *Clostridium_XI* was drastically decreased (*p* < 0.05), while that of *unclassified_Ruminococcaceae* and *Subdivision5_genera_incertae_sedis* were increased in the ileum of goats immunized with the SS vaccine (Figure 3B, *p* < 0.05). Meanwhile, the dominant genera in the cecum were *unclassified_Ruminococcaceae* (5.8%~13.6%), *unclassified_Lachnospiraceae* (7.5%~9.6%), *Clostridium_XI* (4.2%~5.8%), and *Subdivision5_genera_incertae_sedis* (1.0%~3.3%) in three treatments (Figure 3C). These results verify the heterogeneity of the effect of the SS vaccine among GIT microbiomes.

**Figure 4:**
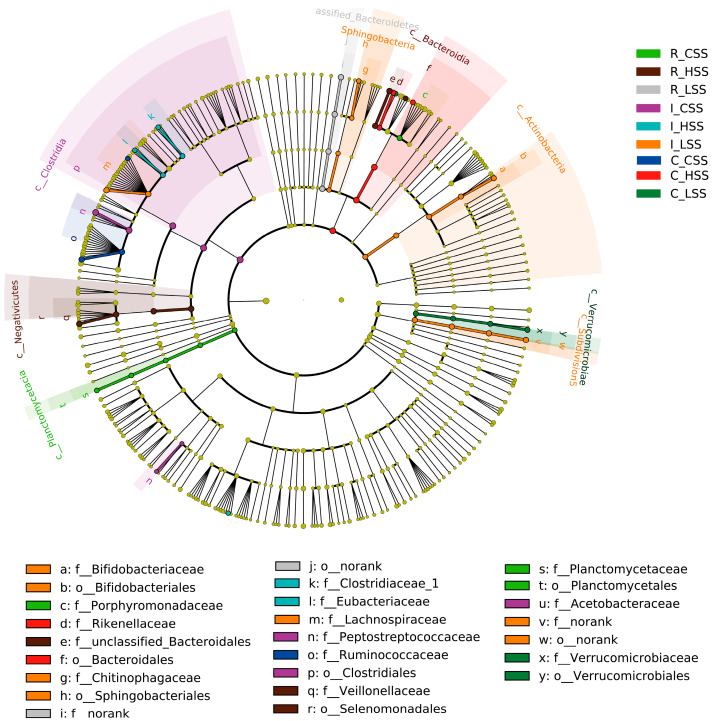
The cladogram by Lefse analysis in GIT of goat vaccinated with different doses of somatostatin. R_CSS: control group in the rumen; I_CSS: control group in the ileum; C_CSS: control group in the cecum; R_LSS: low-dose group in the rumen; I_LSS: low-dose group in the ileum; C_LSS: low-dose group in the cecum; R_HSS: high-dose group in the rumen; I_HSS: high-dose group in the ileum; C_HSS: high-dose group in the cecum.

Further, we found that the microbial biomarkers were associated with vaccine dose in GIT of goat, using the linear discriminant analysis effect size algorithm (Lefse) (Figure 4). Specifically, 25 key differential phylotypes were obtained among three groups of each region; of them, *Rikenella* and *Selenomonadales* were enriched in the L_SS group and the H_SS in the rumen, respectively (LDA scores > 4). In the ileum, the L_SS group selected greater *Lachnospiraceae*, *Actinobacteria*, *Bifidobacteriaceae*, and the H_SS group selected greater *Eubacterium* (LDA scores > 4). Moreover, we identified eight vital phylotypes in response to SS DNA vaccination in the cecum, including *Ruminococcacease*, *Butyrivibrio*, *Akkermansia,* and *Verrucomicrobiae* (LDA scores > 4).

### 3.5. Prediction of the Functional Variation of Microbiota in GIT by SS Vaccination

**Figure 5:**
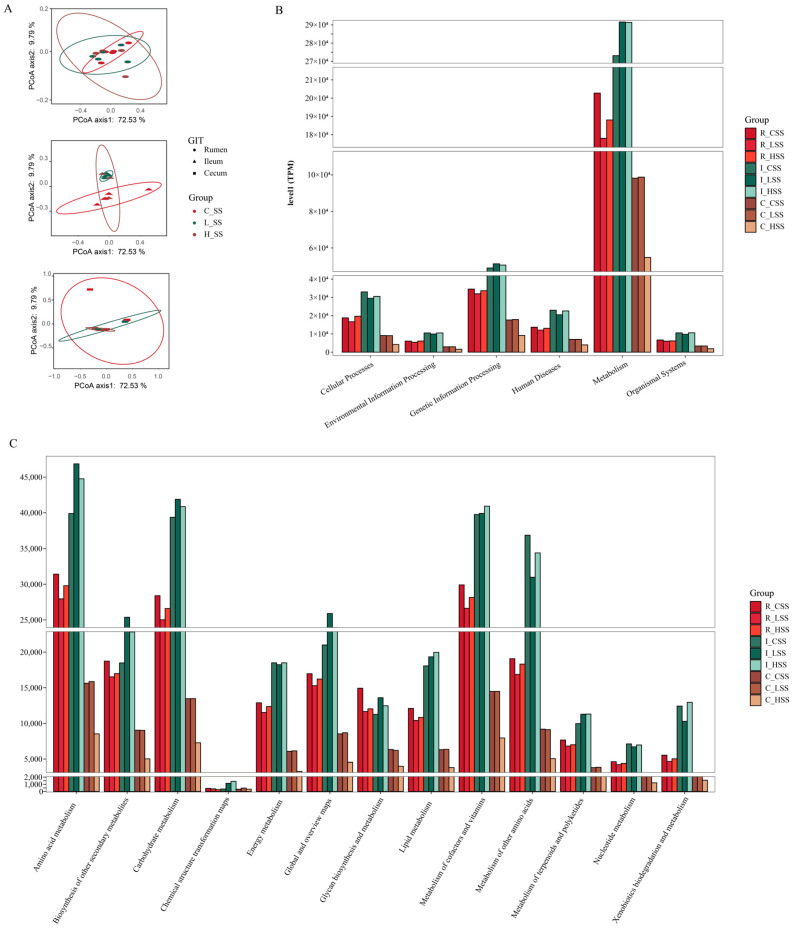
The predicted functions of goats vaccinated with different doses of somatostatin. (**A**) PCoA analysis of KOs profiles. (**B**) Abundance of genes at level 1. (**C**) Abundance of genes at level 2. R_CSS: control group in the rumen; I_CSS: control group in the ileum; C_CSS: control group in the cecum; R_LSS: low-dose group in the rumen; I_LSS: low-dose group in the ileum; C_LSS: low-dose group in the cecum; R_HSS: high-dose group in the rumen; I_HSS: high-dose group in the ileum; C_HSS: high-dose group in the cecum.

**Figure 6:**
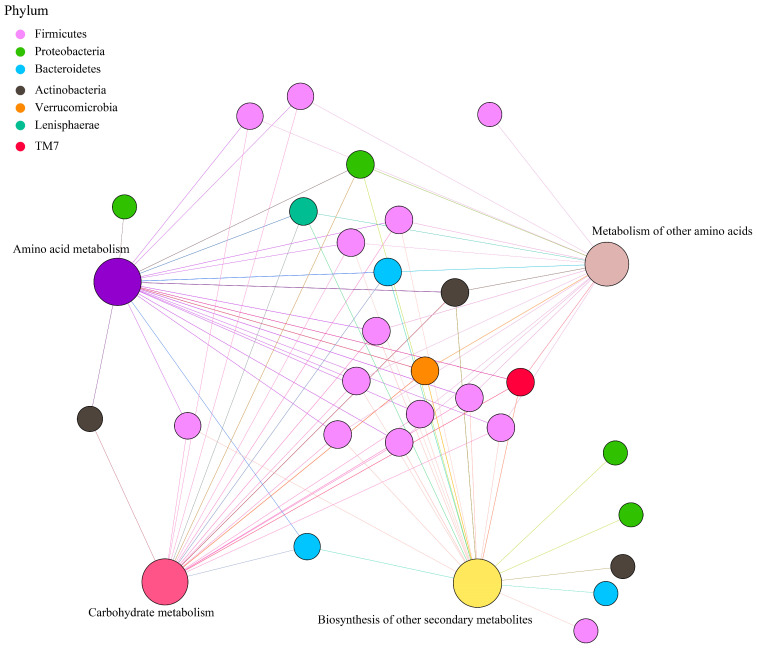
Correlation network of metabolic pathways and genera, with phylum colored according to taxonomic information.

Principal coordinate analysis (PCoA) based on Bray–Curtis distance of microbial genes showed that somatostatin vaccination selected distinct metabolic functions in each GIT region (Figure 5A). Intriguingly, “metabolism pathway” was the most abundant category affected by vaccine dose at level 1 (Figure 5B), in particular for “Amino acid metabolism”, “Carbohydrate metabolism”, “Metabolism of other amino acids”, and “Biosynthesis of other secondary metabolites” (Figure 5C). Further, insights from the key metabolic pathway indicated that 21, 20, 18, and 22 genera were predicated to possess the capabilities of amino acid metabolism, carbohydrate metabolism, metabolism of other amino acids, and biosynthesis of other secondary metabolites, respectively (Figure 6). These genera are affiliated to *Firmicutes* (51.9%), *Proteobacteria* (14.8%), *Bacteroidetes* (11.1%), and *Actinobacteria* (11.1%) at the phylum level.

**Figure 7:**
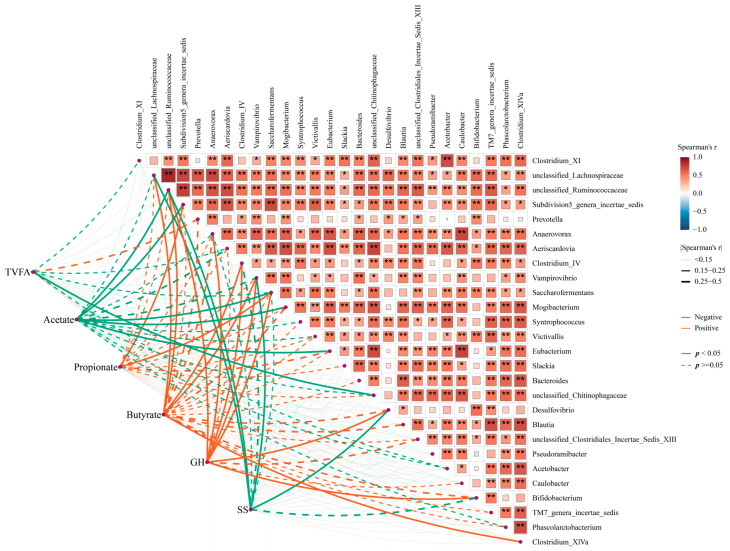
The correlation between functional microorganisms and phenotypes. Orange colors represent negative correlations, whereas green colors represent positive correlations. Asterisks denote Spearman’s significance levels: * *p* < 0.50, ** *p* < 0.01.

Moreover, Spearman’s correlation analysis indicated that these key genera might have a close association with the hormone- and fermentation-related indexes in the GIT of goat. Specifically, the correlation analysis of fermentability indexes and these key genera showed a significant negative correlation between acetate and *Subdivision5_genera_incertae_sedis* and *Saccharofermentans*, while these genera were positively associated with propionate. Similarly, *Clostridium_IV* and *Clostridium_XlVa* were positively associated with butyrate (Figure 7, *p* < 0.05). Additionally, some genera, including *unclassified_Ruminococcaceae*, *Desulfovibrio*, *Bifidobacterium*, *Clostridium_XlVa*, and *Saccharofermentans*, were positively associated with GH, while *unclassified_Ruminococcaceae* and *Desulfovibrio* were negatively associated with SS (*p* < 0.05). Taken together, these data indicate that somatostatin vaccination was accompanied by altering the GIT commensal bacterial structure and function, and, thus, it modified the fermentability and affected hormone level, improving the productivity of the host.

## 4. Discussion

The immunoneutralization of SS is considered an effective method for neutralizing endogenous SS in peripheral blood, promoting the productivity of animals [14]. Analogously, a study in piglets demonstrated that the average daily gain of animals was increased by 32.88% 4 weeks after piglets were administered oral DNA vaccine at a dose of 5 × 10^10^ when compared with the PBS control group [17]. Additionally, a study in ewes revealed that the milk protein and lactose contents of goats were significantly higher in the immunized group than those in the negative control group at week 6 of lactation [30]. Moreover, previous studies on rodents verified that the growth of mice was promoted by immunizations with SS DNA vaccine [31]. Our results reveal that the SS immunization can improve the weight gain in a dose-dependent manner. These phenomena were consistent with the study in piglets, which emphasized that the vaccine induced SS-specific antibodies in a dose-dependent pattern [17]. However, our results show that there was no statistical difference between the high-dose group and the negative control group. The discrepancy in fattening performance between low doses and high doses of the SS DNA oral vaccine may be attributed to species differences in the subjects. Moreover, it is plausible that a negative feedback mechanism may be operative within the goat [32].

As mentioned above, SS is well known as one of the hormones of the GIT [12]. Previously, the SS has been confirmed to regulate animal growth by the GH-IGF-1 axis, and immunization of the SS DNA vaccine can promote animal weight gain [10]. Moreover, our associates found that low-dose SS DNA vaccination improves the growth of goats through both central and peripheral pathways, by manipulating the levels of GH and SS in the serum (Figure S1). Thus, we measured the concentration of GH and SS in GIT to investigate the hormone levels in response to immunoneutralization SS. Undeniably, the concentration of those hormone in GIT was dependent on many factors, including the secretion of endocrine cell, host intake, and transit velocity [33]. Our results showed that the concentration of GH and SS in GIT were affected by the SS vaccine in a dose- and region-dependent manner, which indicates the discrepant influences of gastrointestinal function with different doses of SS vaccine. Combined with the profile of hormone levels in the serum (Figure S1), the profile of the GH concentration in the ileum and cecum of goat when immunized with the SS DNA vaccine may be interpreted as the GH entering and accumulating in the host circulatory system after penetrating the epithelial barrier, where they are sensed by cells, and then eliciting a wide range of biological functions via different receptors and mechanism [34]. More research is needed to explore this mechanism in the future.

It is well known that ruminants are distinguishable from monogastric animals due to their unique digestive system [35], which harbors bacteria, archaea, protozoa, and fungi [36]. The complex gastrointestinal microbial ecosystem has been an immense source of diverse enzymes for plant polysaccharide depolymerization [37], where depolymerized recalcitrant sugar polymers of the plant cell wall are primarily depolymerized into small oligosaccharides [38]. Finally, these oligosaccharides are fermented into short-chain fatty acids (SCFAs) via diverse downstream pathways by most members of the rumen microbiome [4]. Notably, SCFAs are well known as a major energy source for hosts, and directly affect the growth of ruminants [4,20]. Increasing evidence highlights the significance of the GIT microbiota as a central actor in driving beneficial effects of dietary nutrients in animals, with SCFAs as key bacterial metabolites [39]. Of them, butyrate is one of the most important metabolites produced through gastrointestinal microbial fermentation, and possesses enhanced intestinal barrier function and mucosal immunity [40]. Our data show that the concentrations of SCFAs in the rumen were higher in the goat with the low-dose SS DNA vaccine. These results, along with the productivity, indicate that the SS vaccine may enhance the energy generation in the rumen and improve the immunization in the ileum by altering gastrointestinal fermentation.

As mentioned above, the fermentation in the ruminant gastrointestinal tract is due to the complex gastrointestinal microbial ecosystem [37]. Additionally, the SS, known as a common gut–brain peptide, is associate with gut microbiota patterns [41], and the SCFAs are the most important metabolites produced by intestinal flora [42]. Our results suggest that the diversity of the microbiota in the gastrointestinal tract was affected by immunizations with the SS DNA vaccine. In view of the Shannon and Simpson indices indicating microbial community diversity [1], our results imply that the intestinal microbial community diversity was elevated after SS DNA vaccination.

Despite the fact that the *Salmonella* (used in our DNA vaccine) that attaches to *Enterobacteriaceae* was not the dominant strain at the genus level, our results showed that the relative abundances of *Enterobacteriaceae* were 1.95%~6.86% and 2.15%. We suspect that these results may be owing to the database used and the comparison method. Future studies incorporating more advanced techniques, such as metagenome and single-cell microfluidics, are needed to comprehensively analyze the presence and role of *Salmonella* in the host to better understand the microbial composition and its interaction with the vaccine.

Notably, studies have shown that *Rikenella* and *Selenomonadales* are identified as probiotics in the gut [35,43,44]; these were enriched in the L_SS group and H_SS in the rumen in our results. Meanwhile, the abundance of *Clostridium_XI*, well known as a predominant bacteria in the human large intestine [45], was drastically decreased in the rumen, which classified it as one of the important harmful bacteria in the gastrointestine. This indicates that immunizing with the SS DNA vaccine can promote the colonization of beneficial microorganisms and inhibit the colonization of harmful microorganisms in the rumen. Moreover, we found that the L_SS goat selected greater *Lachnospiraceae*, *Actinobacteria*, *Bifidobacteriaceae*, and the H_SS group selected greater *Eubacterium* in the ileum. *Lachnospiraceae* is one of the most abundant and widely occurring bacterial groups in the human gastrointestinal tract [46], and are regarded as volatile metabolite producers and consumers in human feces. Furthermore, *Actinobacteria* are enormously important in human medicine, agriculture, and food production, and key to this is their proven ability to interact with other organisms in the microbial ecosystem [47]. Jami et al. demonstrated the significant diversity of Actinobacteria in the gut microbiota and their potential to produce biologically active compounds [47]. Therefore, our results suggest that these microorganisms are enriched in the ileum, indicting that active immunization of SS also has beneficial effects on the colonization of beneficial bacteria in the small intestine. The colonization of *Ruminococcacease*, *Butyrivibrio*, *Akkermansia,* and *Verrucomicrobiae* in the cecum were altered by SS DNA vaccination. *Ruminococcacease* and *Butyrivibrio* are deemed to possess the digestibility of cellulose and other recalcitrant plant polysaccharides in the hind gut [37]. Another notable phenomenon lies in the increase in *Akkermansia* in the cecum of SS goats. As a typically recognized mucin-degrading bacterium, its increase will be linked to the improved immune function, thereafter eliciting greater animal growth of goats [48]. These microorganisms are deemed to possess digestibility and immunity in the hind gut [37,48]. In short, microbiota capable of converting nutrients show enriched responses to somatostatin vaccination of ruminants, especially in the ileum and the cecum.

Together, goats immunized with the SS DNA vaccine promote the growth performance and may promote the colonization of beneficial microorganisms and inhibit the colonization of harmful microorganisms, changing the type of fermentation in GIT, which alters the strategy of nutrient uptake through the diet.

## 5. Conclusions

We evaluated the efficacy of the somatostatin immunoneutralization on growth, hormone level, and gastrointestinal microbiota of goat, as well as the relationship between those phenotypes and the functional microorganisms with the use of vaccines. Our study extends the understanding of the somatostatin vaccine regulation of ruminants’ growth insight to a gastrointestinal microbial perspective. Despite our extensive studies through the GIT, we are still limited by the boundaries of current technology and insufficient sample size with regard to mucosal or humoral immune responses. Further studies should focus on the more in-depth evaluated omics techniques to illustrate how the mechanism of somatostatin vaccine selection of distinct gastrointestinal microorganisms drives the metabolism process in goats.

## Data Availability

The data that support the findings of this study have been deposited into Sequence Read Archive (SRA) of The National Center for Biotechnology Information (NCBI) with accession number Bio Project: PRJNA1109339.

## Supplementary Materials

The following supporting information can be downloaded at: https://www.mdpi.com/article/10.3390/ani15050728/s1, Figure S1: Anti-SS antibody titres in the different dose somatostatin. Data are expressed as mean ± SEM. Different letters between groups represented significant differences. Figure S2: The somatostatin vaccination changing the hormone levels of serum in goats. A. Linear regression analysis of the concentration of growth hormone (GH) in serum of goats with different dose vaccine; B. Linear regression analysis of the concentration of somatostatin (SS) in serum of goats with different dose vaccine; C. The GH concentration of serum among three groups at week 4, 8, 16 and 20; D. The SS concentration of serum among three groups at week 4, 8, 16 and 20. C_SS: control group; L_SS: low dose group; H_SS: high dose group; Significance was tested using independent one-way Anova or *t*-test. Asterisks denote significant *p* values: NS. *p* > 0.05, * *p* < 0.05, ** *p* < 0.01, n = 5/group. Figure S3: The correlation between alpha diversity indices and specific bacterial taxa. Asterisks denote significant *p* values: NS. *p* > 0.05, * *p* < 0.05, ** *p* < 0.01, *** *p* < 0.001. Figure S4: The profile of dominant family in rumen (A), ileum (B) and cecum (C) of goat vaccinates different dose somatostatin. C_SS: control group; L_SS: low dose group; H_SS: high dose group.
