# Supplementary material for: Insight into the Potential of Somatostatin Vaccination with Goats as a Model: From a Perspective of the Gastrointestinal Microbiota
Source: Animals (Basel). 2025 Mar 4;15(5):728. doi: 10.3390/ani15050728 (PMC11899232; doi:10.3390/ani15050728)
Supplement: Supplementary file 1 [file animals-15-00728-s001.zip › animals-3396125-supplementary.pdf]

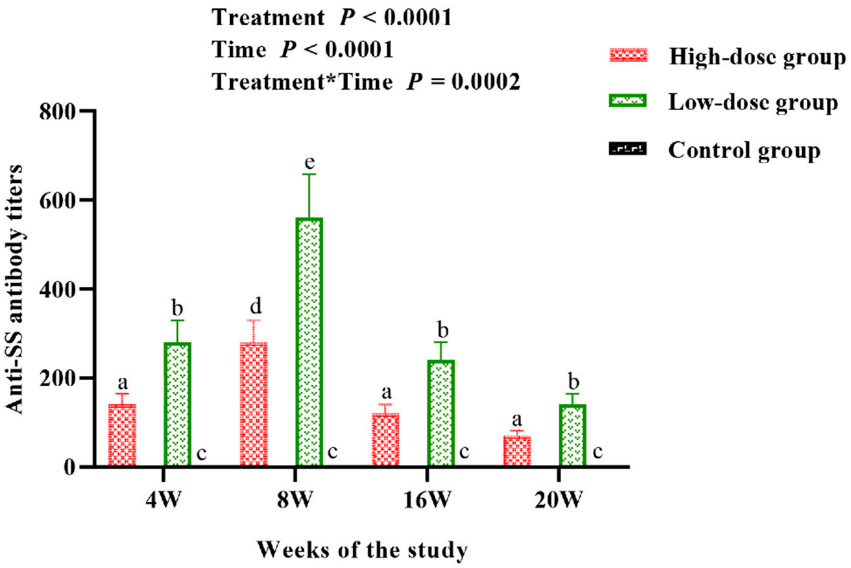


**Figure S1.** Anti-SS antibody titres in the different dose somatostatin. Data areexpressed as mean ±SEM. Different letters between groups represented significant differences.

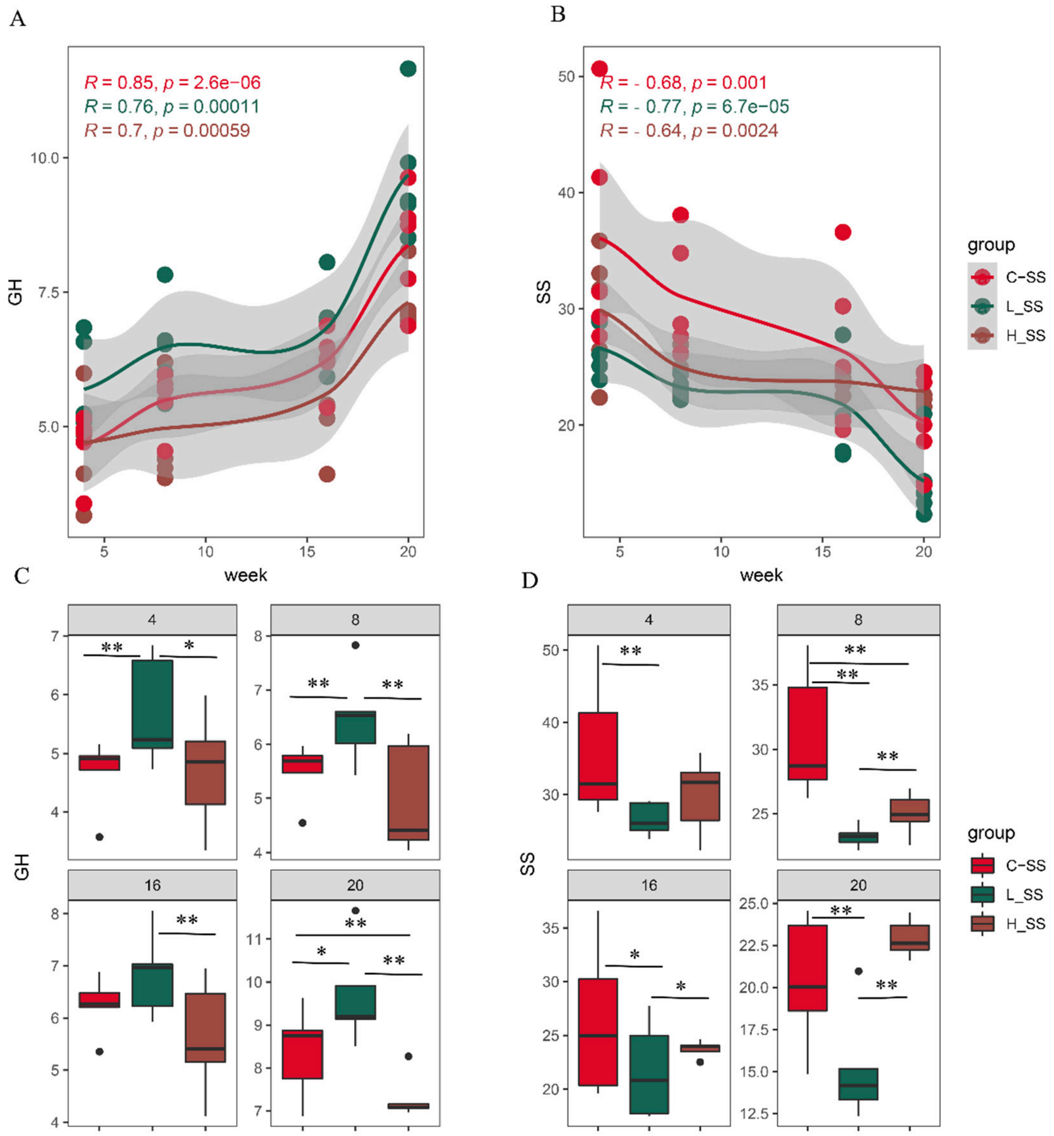


**Figure S2.** The somatostatin vaccination changing the hormone levels of serum in goats. A. Linear regression analysis of the concentration of growth hormone (GH) in serum of goats with different dose vaccine; B. Linear regression analysis of the concentration of somatostatin (SS) in serum of goats with different dose vaccine; C. The GH concentration of serum among three groups at week 4, 8,16 and 20; D. The SS concentration of serum among three groups at week 4, 8,16 and 20. C_SS: control group; L_SS: low dose group; H_SS: high dose group; Significance was tested using independent one-way Anova or T-test. Asterisks denote significant p values: NS. $P > 0.05$, $*P < 0.05$, $**P < 0.01$, n = 5/group.

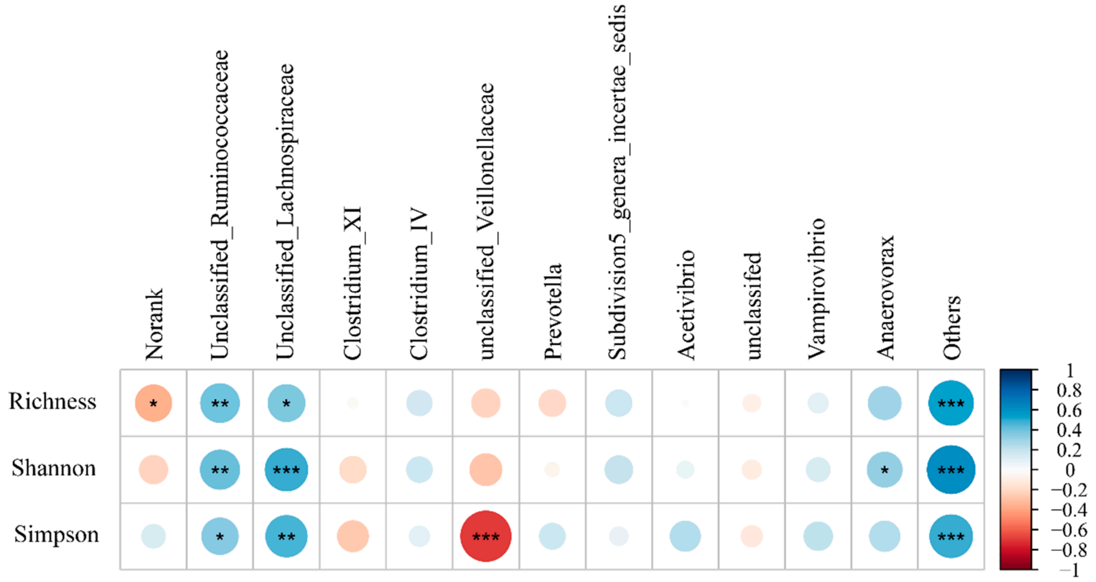


**Figure S3.** The correlation between alpha diversity indices and specific bacterial taxa. Asterisks denote significant p values: NS. $P > 0.05$, *$P < 0.05$, **$P < 0.01$, ***$P < 0.001$.

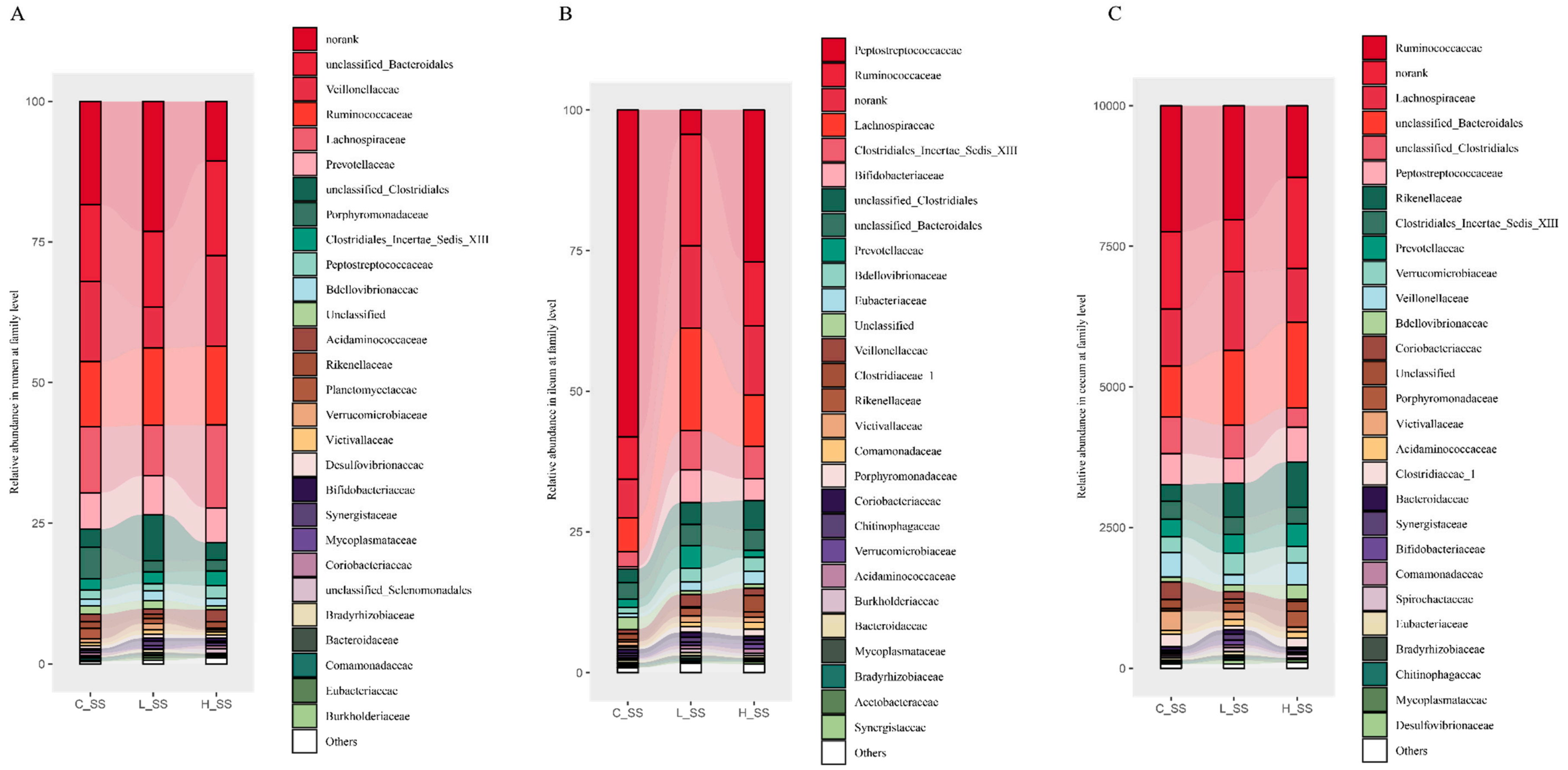


**Figure S4.** The profile of dominant family in rumen (A), ileum (B) and cecum (C) of goat vaccinates different dose somatostatin. C_SS: control group; L_SS: low dose group; H_SS: high dose group.
